# Parkinson’s disease in GTP cyclohydrolase 1 mutation carriers

**DOI:** 10.1093/brain/awu179

**Published:** 2014-07-02

**Authors:** Niccolò E. Mencacci, Ioannis U. Isaias, Martin M. Reich, Christos Ganos, Vincent Plagnol, James M. Polke, Jose Bras, Joshua Hersheson, Maria Stamelou, Alan M. Pittman, Alastair J. Noyce, Kin Y. Mok, Thomas Opladen, Erdmute Kunstmann, Sybille Hodecker, Alexander Münchau, Jens Volkmann, Samuel Samnick, Katie Sidle, Tina Nanji, Mary G. Sweeney, Henry Houlden, Amit Batla, Anna L. Zecchinelli, Gianni Pezzoli, Giorgio Marotta, Andrew Lees, Paulo Alegria, Paul Krack, Florence Cormier-Dequaire, Suzanne Lesage, Alexis Brice, Peter Heutink, Thomas Gasser, Steven J. Lubbe, Huw R. Morris, Pille Taba, Sulev Koks, Elisa Majounie, J. Raphael Gibbs, Andrew Singleton, John Hardy, Stephan Klebe, Kailash P. Bhatia, Nicholas W. Wood

**Affiliations:** 1 Department of Molecular Neuroscience, UCL Institute of Neurology, London WC1N 3BG, UK; 2 IRCCS Istituto Auxologico Italiano, Department of Neurology and Laboratory of Neuroscience – Department of Pathophysiology and Transplantation, “Dino Ferrari” Centre, Università degli Studi di Milano, 20149 Milan, Italy; 3 Department of Neurology, University Hospital, 97080 Würzburg, Germany; 4 Parkinson Institute, Istituti Clinici di Perfezionamento, 20126 Milan, Italy; 5 Sobell Department of Motor Neuroscience and Movement Disorders, UCL Institute of Neurology, London WC1N 3BG, UK; 6 Department of Neurology, University Medical Centre Hamburg-Eppendorf, 20246 Hamburg, Germany; 7 Department of Paediatric and Adult Movement Disorders and Neuropsychiatry, Institute of Neurogenetics, University of Lübeck, 23538 Lübeck, Germany; 8 UCL Genetics Institute, London WC1E 6BT, UK; 9 Neurogenetics Unit, National Hospital for Neurology and Neurosurgery, London WC1N 3BG, UK; 10 Neurology Clinic, Attiko Hospital, University of Athens, 126 42 Haidari, Athens, Greece; 11 Neurology Clinic, Philipps University, 35032 Marburg, Germany; 12 Reta Lila Weston Institute of Neurological Studies, UCL Institute of Neurology, London WC1N 3BG, UK; 13 Division of Inborn Errors of Metabolism, University Children’s Hospital Heidelberg, 69120 Heidelberg, Germany; 14 Institut of Human Genetics, Julius-Maximilian-University, 97070 Würzburg, Germany; 15 Department of Nuclear Medicine, University Hospital, 97080 Würzburg, Germany; 16 Department of Nuclear Medicine, Fondazione IRCCS Ca’ Granda Ospedale Maggiore Policlinico, 20122 Milano, Italy; 17 Serviço de Neurologia, Hospital Beatriz Ângelo, 2674-514 Loures, Portugal; 18 Movement Disorder Unit, CHU Grenoble, Joseph Fourier University, and INSERM U836, Grenoble Institute Neuroscience, F-38043 Grenoble, France; 19 Université Pierre et Marie Curie-Paris6, Centre de Recherche de l'Institut du Cerveau et de la Moelle épinière, UMR-S975; Inserm, U975, Cnrs, UMR 7225, Paris, France; 20 Centre d'Investigation Clinique (CIC-9503), Département de Neurologie, Hôpital Pitié-Salpétriêre, AP-HP, Paris, France; 21 Département de Génétique et Cytogénétique, Pitié-Salpêtrière hospital, 75013 Paris, France; 22 DZNE–Deutsches Zentrum für Neurodegenerative Erkrankungen (German Centre for Neurodegenerative Diseases), Hertie Institute for Clinical Brain Research, University of Tübingen, 72076 Tübingen, Germany; 23 Department of Clinical Neuroscience, UCL Institute of Neurology, London WC1N 3BG, UK; 24 Department of Neurology and Neurosurgery, University of Tartu, 50090 Tartu, Estonia; 25 Department of Pathophysiology, Centre of Excellence for Translational Medicine, University of Tartu, 50411 Tartu, Estonia; 26 Laboratory of Neurogenetics, National Institute on Aging, Bethesda, MD 20892, USA

**Keywords:** *GCH1*, DOPA-responsive-dystonia, Parkinson’s disease, dopamine, exome sequencing

## Abstract

GTP cyclohydrolase 1, encoded by the *GCH1* gene, is an essential enzyme for dopamine production in nigrostriatal cells. Loss-of-function mutations in *GCH1* result in severe reduction of dopamine synthesis in nigrostriatal cells and are the most common cause of DOPA-responsive dystonia, a rare disease that classically presents in childhood with generalized dystonia and a dramatic long-lasting response to levodopa. We describe clinical, genetic and nigrostriatal dopaminergic imaging ([^123^I]N-ω-fluoropropyl-2β-carbomethoxy-3β-(4-iodophenyl) tropane single photon computed tomography) findings of four unrelated pedigrees with DOPA-responsive dystonia in which pathogenic *GCH1* variants were identified in family members with adult-onset parkinsonism. Dopamine transporter imaging was abnormal in all parkinsonian patients, indicating Parkinson’s disease-like nigrostriatal dopaminergic denervation. We subsequently explored the possibility that pathogenic *GCH1* variants could contribute to the risk of developing Parkinson’s disease, even in the absence of a family history for DOPA-responsive dystonia. The frequency of *GCH1* variants was evaluated in whole-exome sequencing data of 1318 cases with Parkinson’s disease and 5935 control subjects. Combining cases and controls, we identified a total of 11 different heterozygous *GCH1* variants, all at low frequency. This list includes four pathogenic variants previously associated with DOPA-responsive dystonia (Q110X, V204I, K224R and M230I) and seven of undetermined clinical relevance (Q110E, T112A, A120S, D134G, I154V, R198Q and G217V). The frequency of *GCH1* variants was significantly higher (Fisher’s exact test *P*-value 0.0001) in cases (10/1318 = 0.75%) than in controls (6/5935 = 0.1%; odds ratio 7.5; 95% confidence interval 2.4–25.3). Our results show that rare *GCH1* variants are associated with an increased risk for Parkinson’s disease. These findings expand the clinical and biological relevance of GTP cycloydrolase 1 deficiency, suggesting that it not only leads to biochemical striatal dopamine depletion and DOPA-responsive dystonia, but also predisposes to nigrostriatal cell loss. Further insight into *GCH1*-associated pathogenetic mechanisms will shed light on the role of dopamine metabolism in nigral degeneration and Parkinson’s disease.

## Abstract (precis)

Mutations in the gene encoding the dopamine-synthetic enzyme GTP cyclohydrolase-1 (*GCH1*) cause DOPA-responsive dystonia (DRD). Mencacci *et al.* demonstrate that *GCH1* variants are associated with an increased risk of Parkinson's disease in both DRD pedigrees and in patients with Parkinson's disease but without a family history of DRD.

## Introduction

Parkinson’s disease is a common neurodegenerative disease mainly characterized by severe loss of dopaminergic neurons in the substantia nigra pars compacta and by the formation of α-synuclein positive aggregates (Lees *et al.*, 2009). Nigral neuron degeneration and consequent decrease in dopaminergic striatal innervation result in classic Parkinson’s disease motor symptoms. Symptomatic treatment with levodopa or dopamine agonists is effective in alleviating these symptoms, although, along with disease progression, levodopa-induced motor complications (e.g. dyskinesias, wearing-off, on-off fluctuations) may appear.

In recent years several Mendelian loci have been unequivocally linked to hereditary forms of Parkinson’s disease (Houlden and Singleton, 2012) and genome-wide association studies have succeeded in identifying many common, low risk variants (Plagnol *et al.*, 2011).

The *GCH1* gene (14q22.1-q22.2; OMIM 600225) encodes GTP cyclohydrolase 1, the enzyme controlling the first and rate-limiting step of the biosynthesis of tetrahydrobiopterin (BH_4_), the essential cofactor for the activity of tyrosine hydroxylase, and for dopamine production in nigrostriatal cells (Kurian *et al.*, 2011). Mutations in *GCH1* are the most common cause of DOPA-responsive dystonia (DYT5; OMIM#128230) (Clot *et al.*, 2009), a rare movement disorder that presents typically in childhood with lower limb dystonia and subsequent generalization (Nygaard, 1993*b*). The hallmark of the disease is an excellent and sustained response to small doses of levodopa, generally without the occurrence of motor fluctuations (Trender-Gerhard *et al.*, 2009). Reduction of CSF levels of pterins, dopamine and serotonin metabolites (Assmann *et al.*, 2003), or an abnormal phenylalanine-loading test (Bandmann *et al.*, 2003) are supportive findings in the diagnosis of DOPA-responsive dystonia. Inheritance is usually autosomal dominant with incomplete penetrance (Furukawa *et al.*, 1998), though recessive cases have been described (Opladen *et al.*, 2011). Dominant *GCH1* mutations result in a significant reduction of GCH1 activity through a dominant negative effect of the mutant protein on the normal enzyme (Hwu *et al.*, 2000).

Neuropathological examination in a limited number of cases with DOPA-responsive dystonia, revealed marked reduction of melanin pigment and dopamine content in nigrostriatal neurons, but no evidence of nigral cell loss or degeneration (Furukawa *et al.*, 1999).

Parkinsonian features are frequently detected in patients with DOPA-responsive dystonia (Tassin *et al.*, 2000) and family studies have shown that carriers of *GCH1* mutations may develop adult-onset parkinsonism in the absence of dystonia (Nygaard *et al.*, 1990). Based on previous studies, the prevailing hypothesis was that parkinsonism represented an atypical, age-specific, presentation of DOPA-responsive dystonia without nigral degeneration (Nygaard and Wooten, 1998).

The aim of this study was to further explore the relationship between *GCH1* mutations and parkinsonism and consider whether adult *GCH1* mutation carriers are at increased risk of developing neurodegenerative Parkinson’s disease.

We first describe the clinical, genetic and nigrostriatal dopaminergic imaging findings of DOPA-responsive dystonia pedigrees in which pathogenic *GCH1* variants were identified in family members with adult-onset parkinsonism. We subsequently explore the hypothesis that *GCH1* variants might be associated with an increased risk for Parkinson’s disease, even without a family history for DOPA-responsive dystonia, through examination of whole-exome sequencing data from a large cohort of cases and controls.

## Materials and methods

### Family study

#### Pedigrees

The clinical and demographic features of the pedigrees with *GCH1* mutations involved in this study are described in the ‘Results’ section. DOPA-responsive dystonia pedigrees were included in the study, where family members affected with adult-onset parkinsonism were available for clinical and genetic examination and in whom dopaminergic studies had been performed. Local ethics committees approved the study and informed consent for genetic testing was obtained in all cases.

#### Genetic analysis

Genomic DNA was extracted from peripheral blood leucocytes using standard procedures. Probands were screened for *GCH1* mutations (NCBI transcript NM_000161.2) by standard bi-directional Sanger sequencing of all six coding exons and exon-intron boundaries (primer sequences available on request). Dosage analysis for *GCH1* exonic deletions and duplications was performed by multiplex ligation-dependent probe amplification (MLPA, MRC).

#### Dopamine transporter imaging studies

Dopaminergic striatal innervation was evaluated as dopamine reuptake transporter (DAT) density by means of single photon computed tomography (SPECT) and [^123^I]N-ω-fluoropropyl-2β-carbomethoxy-3β-(4-iodophenyl) tropane (^123^I-FP-CIT). SPECT data acquisition and reconstruction has been described in detail elsewhere (Isaias *et al.*, 2010). To obtain comparable measurements among different centres, ^123^I-FP-CIT binding values for the caudate nucleus and putamen were calculated by means of the basal ganglia matching tool (Nobili *et al.*, 2013).

### Whole-exome sequencing study

#### Participants and study design

The study initially involved 1337 unrelated subjects with Parkinson’s disease and 1764 control subjects of European origin or North American of European descent that underwent whole-exome sequencing. Cases, originating mainly from the USA, UK, Holland and France, were recruited by the International Parkinson Disease Genomics Consortium (IPDGC), an international collaboration to understand the genetics of Parkinson’s disease.

A further 190 cases with Parkinson’s disease were recruited through a community-based epidemiological study of Parkinson’s disease in Estonia (University of Tartu, Estonia). Cases with Parkinson’s disease were clinically diagnosed according to the UK Parkinson’s Disease Society Brain Bank (UKPDSBB) criteria (Hughes *et al.*, 1992).

Control samples were collected by the UCL-exomes, a consortium of researchers within University College London (London, UK) designed to share raw read level data from multiple exome sequencing projects. Control subjects had no diagnosis of Parkinson’s disease, DOPA-responsive dystonia or any other movement disorder. Whole-exome sequencing data from an additional 4300 North American individuals of European descent were analysed from the publicly available NHLBI Exome Sequencing Project Exome Variant Server (EVS) database (http://evs.gs.washington.edu/EVS/).

#### Procedures

Paired-end sequence reads (TruSeq chemistry sequenced on the Illumina HiSeq 2000) were aligned with Burrows-Wheeler Aligner (for IPDGC) and novoalign (for UCL-exomes) against the reference human genome (UCSC hg19). Duplicate read removal, format conversion, and indexing were performed with Picard (http://picard.sourceforge.net/). The Genome Analysis Toolkit was used to recalibrate base quality scores, perform local realignments around possible indels, and to call and filter the variants. ANNOVAR software was used to annotate the variants (Wang *et al.*, 2010).

Pathogenicity of the identified missense variants was predicted using the following bioinformatics tools: HumVar-trained PolyPhen-2 model (http://genetics.bwh.harvard.edu/pph2/), SIFT (http://sift.jcvi.org/), LRT (s.wustl.edu/jflab/lrt_query.html) and MutationTaster (http://www.mutationtaster.org/). Evolutionary conservation of the mutated amino acids was evaluated using ClustalW2 (http://www.ebi.ac.uk/Tools/msa/clustalw2/).

#### Statistical analysis

Frequencies of coding and splice-site *GCH1* variants in cases and controls were compared by means of Fisher’s exact (statistical significance set at *P*-value < 0.05 using a two-tailed test) and odds ratios (OR) and 95% confidence intervals (CI) were calculated. Analyses were performed using the statistical analysis program R (http://www.r-project.org/).

## Results

### Family study

#### Family A

**Figure 1:**
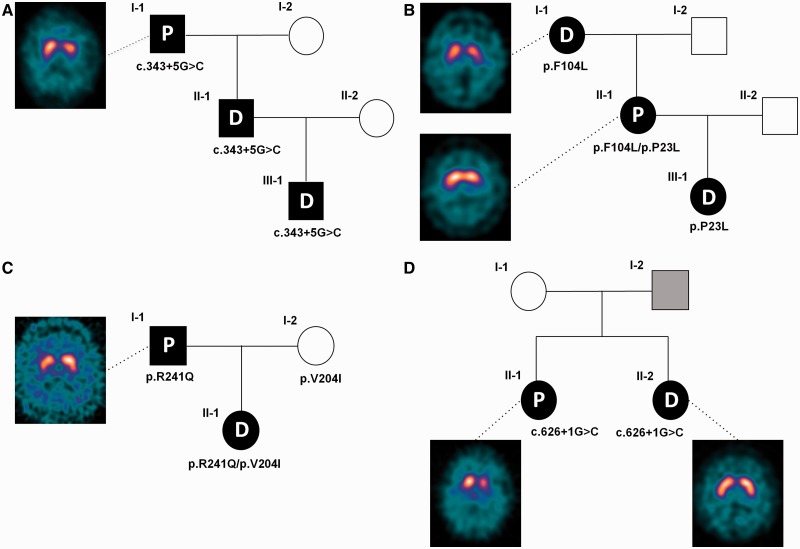
Pedigrees and ^123^I-FP-CIT SPECT scan images of the four families with *GCH1* mutations involved in this study. Subject I-2 of Family D was reported to be affected by a movement disorder (hand tremor) but was not available for clinical or genetic assessment. P = Parkinson’s disease; D = DOPA-responsive dystonia.

The proband (Case III-1, Fig. 1A) is a British 18-year-old male who had a difficult caesarean birth, with perinatal distress and subsequent developmental delay. At 18 months he developed inward turning of his feet with walking difficulties and frequent falls. He was diagnosed clinically with DOPA-responsive dystonia at the age of 3 years and administration of levodopa (300 mg/day) markedly improved his symptoms. [^123^I]FP-CIT SPECT, performed at age 17, was normal (data not shown).

The proband’s father (Case II-1), who was initially thought to have cerebral palsy due to a birth injury, was subsequently diagnosed, at the age of 42, with DOPA-responsive dystonia. The proband’s grandfather (Case I-1) is a 65 year-old male with a 6-year history of progressive asymmetric rest tremor in the right upper limb. Examination showed signs of typical Parkinson’s disease with hypomimia, unilateral rest tremor and asymmetric bradykinesia. He did not present signs of dystonia. ^123^I-FP-CIT SPECT showed bilateral reduced tracer uptake more marked on the left (Fig. 1A), consistent with nigrostriatal dopaminergic denervation. He responded well to levodopa therapy (300 mg/day).

*GCH1* analysis revealed a heterozygous splice site mutation (c.343+5G>C) in the three affected individuals. We previously detected c.343+5G>C in a recessive pedigree, carried by the unaffected mother of two very severely affected children who also inherited the K224R mutation from their unaffected father (Bandmann *et al.*, 1996*b*; Trender-Gerhard *et al.*, 2009). However the c.343+5G>C mutation has not been previously described in DOPA-responsive dystonia dominant pedigrees, making its pathogenicity uncertain. Complimentary DNA analysis showed aberrant splicing resulting in a premature stop codon and retention of intron 1 in a proportion of mutant transcripts, confirming the loss-of-function effect of the variant. See Supplementary material for details of the RNA analysis.

#### Family B

The proband (Case III-1; see Fig. 1B) is a 12-year-old right-handed female of German origin with DOPA-responsive dystonia, with an onset at age 11, with writing and foot dystonia. Her mother (Case II-1) presented at age 39 with progressive loss of dexterity and slowness in her right arm and dystonic posturing of the right foot. Examination showed an asymmetric rigid-akinetic parkinsonian syndrome without tremor and severe right foot fixed dystonia. Levodopa therapy resulted in marked improvement of both dystonic and parkinsonian symptoms. ^123^I-FP-CIT SPECT revealed an asymmetric bilateral reduced tracer uptake, more marked in the left striatum. There was sustained response to levodopa therapy although there was an increase in dose requirement (up to 800 mg/day). Levodopa-induced dyskinesias developed 6 years after initiation of levodopa. Examination of the proband’s 66-year-old grandmother (Case I-1) revealed oromandibular dyskinesias and upper limb dystonic features. She declined a trial of levodopa. Her ^123^I-FP-CIT SPECT displayed border-line reduced DAT values in both putamens.

*GCH1* screening in this family revealed two variants: c.68C>T;p.P23L (carried by Cases III-1 and II-1) and c.312C>A;p.F104L (carried by Cases II-1 and I-1). There were no *GCH1* exonic rearrangements. F104L is absent in public control data sets and has been previously reported in association with DOPA-responsive dystonia (Clot *et al.*, 2009). P23L (rs41298432) is a benign polymorphism present in population controls at a frequency of 1–2% (Jarman *et al.*, 1997; Hauf *et al.*, 2000).

To confirm GCH1 deficiency, phenylalanine-loading test (100 mg/kg) was performed in Cases I-I and II-I and showed pathologically elevated phenylalanine/tyrosine ratios in both (Supplementary Fig. 2). CSF analysis, performed in Case III-I, displayed low levels of BH_4_ (13 nmol/l; 18–53 nmol/l) and neopterin (6 nmol/l; 10–31 nmol/l), consistent with GCH1 deficiency. Given the benign nature of P23L, we hypothesize that the GCH1 deficiency confirmed in this patient may be the result of an—as yet—unidentified non-coding causative mutation.

#### Family C

The proband (Case II-1, Fig. 1C) is a German 41-year-old female, affected by DOPA-responsive dystonia, who presented at age 4 years with bilateral foot inversion on walking. Her father (Case I-1) is a 67-year old male with a 1-year history of typical Parkinson’s disease with left hand rest tremor, bilateral rigidity and bradykinesia and mild gait difficulties. There was no dystonia. ^123^I-FP-CIT SPECT examination revealed asymmetrically reduced DAT-density in the striatum. Rasagiline and pramipexole were started with good response. The mother (Case I-2), aged 62 years, had a normal neurological examination.

The proband was compound heterozygous for two *GCH1* missense variants, c.610G>A;p.V204I, inherited from the asymptomatic mother, and the novel variant c.722G>A;p.R241Q, which was paternally inherited. R241Q is absent in public control data sets, is predicted deleterious by all *in silico* prediction tools and involves an amino acid residue conserved down to invertebrate species. Furthermore a pathogenic mutation at the same residue has already been reported (Bandmann *et al.*, 1998).

CSF analysis in the parkinsonian case supported a pathogenic effect of the R241Q mutation on GCH1 activity: pterin analysis revealed low BH_4_ (8 nmol/l; 18–53), but normal neopterin (24 nmol/l; 10–31); neurotransmitter analysis showed low homovanillic acid (95 nmol/l; 115–455) and 5-hydroxyindolacetic acid (59 nmol/l; 61–204), which are metabolites of dopamine and serotonin, respectively.

#### Family D

The proband is an Italian 58-year-old female (Case II-1, Fig. 1D), who developed progressive tremor and clumsiness in the right arm at age 44 years. Clinical examination showed typical Parkinson’s disease with hypomimia, hypophonia and asymmetrical bradykinesia and rigidity. Action dystonic tremor (right > left), poor postural reflexes and slow gait were also evident and there was a sustained response to levodopa. The dose was gradually increased up to 400 mg/day, after which rotigotine 4 mg/day was added. Dyskinesias and wearing-off symptoms developed 6 years after levodopa initiation. ^123^I-FP-CIT SPECT revealed asymmetrically reduced DAT binding values in the striatum.

Her sister (Case II-2; Fig. 1D), aged 60, had a childhood onset of mild walking difficulties. At age 55, she developed exercise-induced left foot dystonia and dystonic tremor in both arms. She had no bradykinesia or other parkinsonian signs. Low-dose levodopa (100 mg alternate days) was started with excellent symptom control. ^123^I-FP-CIT SPECT was normal. Their father was reported to have a tremulous condition, but was not available for clinical or genetic examination. *GCH1* sequencing revealed that both sisters were heterozygous for the previously reported pathogenic mutation c.626+1G>C (Garavaglia *et al.*, 2004).

The main clinical features of the *GCH1* mutation carriers with adult-onset parkinsonism and abnormal ^123^I-FP-CIT SPECT imaging are summarized in Table 1. Their clinical features fully met the UKPDSBB criteria for definite Parkinson’s disease diagnosis. None of these cases presented significant diurnal fluctuations, worsening of symptoms in the evening or substantial sleep benefit, features often recognized in cases with DOPA-responsive dystonia (Kurian *et al.*, 2011). DAT binding values are reported in Supplementary Table 1.

**Table 1:** Characteristics of parkinsonian cases with *GCH1* pathogenic variants and abnormal dopaminergic imaging described in this study and present in the literature

Origin	Sex/age at scan/age at onset (y)	Mutation	Relatives with DRD	Age at levodopa start (y)	Current treatment dose (mg/day)	Parkinsonian features	H&Y score	Dystonic features	Levodopa-induced complications	Scan result	Reference
UK	M/65/59	c.343+5G>C/w	Son and grandson	60	l-DOPA 300	Hypomimia, R hand rest and re-emergent postural tremor, and bilateral rigidity and bradykinesia (R>L)	2	No	No	Bilateral (L>R) reduced DAT density	Present study (Family A)
Germany	F/47/39	F104L/ P23L	Daughter and mother	41	l-DOPA 800	Hypomimia, bilateral rigidity, bradykinesia, reduced arm swinging (R>L), and mild gait difficulties	2	R foot dystonia	Dyskinesias after 6 y of therapy	Bilateral (L>R) reduced DAT density	Present study (Family B)
Germany	M/67/66	R241Q/w	Daughter	/	Rasagiline 1 Pramipexole 0.375	Hypomimia, L hand rest tremor, bilateral bradykinesia and rigidity (L>R), and mild gait difficulties	2	No	No	Bilateral (R>L) reduced DAT-density	Present study (Family C)
Italy	F/58/44	c.626+1 G>C/w	Sister	53	l-DOPA 400 Rotigotine 4	Hypomimia, bilateral rigidity and bradykinesia (R>L), mild postural instability, and gait difficulties	2	Bilateral (R>L) upper limb dystonic tremor	Dyskinesias after 6 y of therapy	Bilateral (L>R) reduced DAT density	Present study (Family D)
Japan	M/54/39	R184H/w	No	40	l-DOPA 600	Cogwheel rigidity, akinesia, and postural instability	NA	Dystonic posture in the four limbs (R>L)	Wearing-off and dyskinesias after 10 y of therapy	Bilateral reduced FD intake	Kikuchi *et al.*, 2004
Denmark	M/38/28	P199S/w	Brother	33	l-DOPA 350 Entacapone Selegiline 5	Bradykinesia and rigidity in the L arm	NA	Dystonia of neck, trunk and four limbs, action tremor (L>R)	Dyskinesias after 2 y of therapy	Bilateral (R>L) reduced DAT density	Hjermind *et al.*, 2006
Germany	F/65/50	Complete deletion of the *GCH1* gene/w	Daughter	60 (for 10 y on dopamine agonist only)	l-DOPA 200 Selegiline 5	Tremor in the R hand, reduced dexterity and mild gait disturbance	NA	No	No	Bilateral (L>R) reduced DAT density	Eggers *et al.*, 2012
Italy	M/59/NA	Deletion of exons 5-6/w	Son with DRD, sister with MSA	NA	NA	Hypomimia, L hand rest tremor. bradykinesia (L>R), mild gait difficulties	NA	No	Dyskinesias after 10 y of therapy	Bilateral reduced DAT density	Ceravolo *et al.*, 2013

NA = not available; DRD = DOPA-responsive dystonia; H&Y = Hoehn and Yahr; F = female; M = male L = left; R = right; MSA = multiple system atrophy; y = years; w = wild-type.

### Whole-exome sequencing study

We hypothesized that pathogenic variants in *GCH1* could be found in subjects with Parkinson’s disease without a family history for DOPA-responsive dystonia. To investigate this we examined whole-exome sequencing data of a large cohort of patients predominantly affected by early-onset or familial Parkinson’s disease and controls. After quality control checks (removal of gender mismatches, duplicate, related and non-Caucasian samples, samples with low call rate or excess of heterozygosity), 1318 cases with Parkinson’s disease and 1635 controls remained. Additional control data (*n* = 4300) were obtained from the publically available Exome Variant Server (EVS) data set.

In total 1318 cases and 5935 controls were analysed for the presence of *GCH1* coding (including small insertions/deletions, missense and stop-gain changes) or splice-site variants (± 5 base pairs from the coding exons). The mean age of subjects with Parkinson’s disease was 55.7 ± 13.9 years (range 17–101; data available for 970 cases) and the mean age at onset was 46.7 ± 13.8 years (range 6–98; data available for 1194 cases). Four hundred and twenty-three of 1194 (35.4%) were early-onset cases (age at onset ≤ 40 years) and ∼630 were familial cases (positive family history for Parkinson’s disease in a first or second-degree relative).

Coverage of the six *GCH1* coding exons (NCBI transcript NM_000161.2) was comparable in the three data sets (IPDGC, UCL-ex and EVS; Supplementary Table 2). No common variants (frequency >1%) were identified. The benign polymorphisms P23L (rs41298432) and P69L (rs56127440), detected at similar frequencies in cases and controls, were not included in the analysis.

The main results of *GCH1* analysis are summarized in Table 2. Combining cases and controls, 11 unique heterozygous *GCH1* variants (10 missense and one stop-gain mutation) were identified in 16 individuals. Six variants were found only in cases with Parkinson’s disease (Q110X, Q110E, A120S, D134G, G217V and M230I), three in controls alone (T112A, I154V and R198Q) and two were detected in both groups (V204I, K224R). The frequency of *GCH1* variants was significantly higher in cases with Parkinson’s disease (10/1318; 0.75%) than in individual (UCL-ex controls 1/1635; 0.06%; *P* = 0.003; OR 12.4 95% CI 1.7–541.1; EVS database 5/4300; 0.11%; *P* = 0.0004; OR 6.5, 95% CI 2.0–24.5) and combined data sets of controls (6/5935; 0.1%; *P* = 0.0001; OR 7.5, 95% CI 2.4–25.3).

**Table 2:** List of *GCH1* variants identified by exome sequencing in patients with Parkinson disease and controls*^a^*

Mutation	Exon	dbSNP	Prediction score^b^	Previously described in DRD?	PD patients (*n* = 1318)	UCL-ex controls (*n* = 1635)	OR (95% CI)	*P*-value	EVS controls (*n* = 4300)	OR (95% CI)	*P*-value	Total controls (*n* = 5935)	OR (95% CI)	*P*-value
All variants					10 (0.75%)	1 (0.06%)	12.4 (1.7–541.1)	0.003	5 (0.11%)	6.5 (2.0–24.5)	0.0004	6 (0.1%)	7.5 (2.4–25.3)	0.0001
c.328C>T; p.Q110X	1		NA	Yes, in dominant and recessive pedigrees	1	0			0			0		
c.328C>G; p.Q110E	1		2	No	1	0			0			0		
c.334A>G; p.T112A	2	rs199990434	2	No	0	0			1			1		
c.358G>T; p.A120S	2		4	No	1	0			0			0		
c.401A>G; p.D134G	2		4	No	1	0			0			0		
c.460A>G; p.I154V	3		2	No	0	1			0			1		
c.593G>A; p.R198Q	5	rs201238926	0	No	0	0			1			1		
c.610G>A; p.V204I	5	rs200891969	4	Yes, in sporadic and recessive cases	3	0			1			1		
c.650G>T; p.G217V	6		4	No	1	0			0			0		
c.671A>G; p.K224R	6	rs41298442	2	Yes, in dominant and recessive pedigrees	1	0			2			2		
c.690G>A; p.M230I	6		4	Yes, in a sporadic case	1	0			0			0		

NA = not applicable; DRD = DOPA-responsive dystonia; PD = Parkinson disease; UCL-ex = University College of London exomes consortium; EVS = Exome Variant Server.

*P-*values were calculated by means of Fisher’s exact test.

^a^ NCBI transcript NM_000161.2.^.^This count includes all detected coding and splice-site variants at any frequency, but the two benign variants P23L and P69L.

^b^ This score, ranging from 0 to 4, indicates the number of tools (Polyphen-2, SIFT, LRT and MutationTaster) predicting a pathogenic effect on the protein function.

All carriers of variants in *GCH1* were negative for pathogenic mutations in the known genes associated with Mendelian forms of parkinsonism (*SNCA*, *LRRK2*, *VPS35*, *PARK2*, *PARK7*, *PINK1*, *ATP13A2*, *PLA2G6* and *FBXO7*). The presence of copy number variants in the *SNCA*, *PARK2*, *PARK7*, and *PINK1* genes was excluded by MLPA in all cases.

One case was heterozygous for the *GBA* mutation E326K. This is a relatively common variant (∼1–2% Caucasians) that was recently shown to be associated with a modest but significant increase in the disease risk (Duran *et al.*, 2013). The main features of the 10 cases with Parkinson’s disease with pathogenic or possibly pathogenic *GCH1* variants are listed in Table 3.

**Table 3:** Clinical features of Parkinson disease cases with *GCH1* variants identified in the exome-sequencing study

Case	Origin	Sex/age/age at onset (y)	*GCH1* mutation	Family history of PD	Age at l-DOPA start (y)	Current treatment (mg/day)	Parkinsonian features	l-DOPA responsi-veness	H&Y score	Cognitive symptoms	Other non-motor features	Dystonia	Levodopa-induced complications
1	USA	F/47/43	M230I/w	No	/	Pramipexole 0.75	Asymmetric onset, bilateral involvement with rest and postural tremor, bradykinesia and rigidity, mild gait difficulties	Good	2	No	No	No	No
2	USA	M/55/37	K224R/w	Yes (father)	NA	NA	Asymmetric onset, moderate bilateral involvement with rest tremor, bradykinesia and rigidity, postural instability and gait difficulties	Good	3	No	Fatigue	No	Dyskinesias and wearing-off
3	Holland	M/49/35	G217V/w	No	43	l-DOPA 600 Tolcapone 400 Pramipexole 3	Asymmetric onset, slurred speech, mild L arm rest and postural tremor, moderate bilateral bradykinesia and rigidity, postural instability	Good	3	Subjective loss of memory (MMSE 29/30)	Hyposmia, ICD	No	Initial dyskinesias and wearing-off
4	UK	M/63/32	V204I/w	Yes (1st degree cousin)	36	DBS, l-DOPA 200 Amantadine 100 Rotigotine 8	Asymmetric onset, hypomimia, slurred speech, hypophonia, marked bilateral rest and postural tremor, moderate bilateral rigidity and bradykinesia, postural instability	Good	4	No	Hyposmia, constipation, RBD	Right foot exercise-induced dystonia at onset	Disabling dyskinesias and on-off fluctuations
5	Estonia	M/75/61	V204I/w	Yes (mother)	61	l-DOPA 400 Pramipexole 3.15	Asymmetric onset, rest and postural tremor (R>L), bradykinesia and rigidity, mild gait disorder, hypomimia	Good	3	Mild cognitive impairment	Hyposmia, fatigue, sleep and bladder disorder	Lower limb off-dystonia	On-off fluctuations (30% of waking day in off-state)
6	Estonia	M/72/59	V204I/w	Yes (mother)	65	l-DOPA 600 Entacapone 800	Asymmetric onset, bilateral bradykinesia and rigidity (L>R), no tremor. Mild gait difficulties and postural instability	Good	3	No	Hyposmia, sleep and bladder disorder	No	Dyskinesias (30-40% of waking day), wearing-off
7	USA	M/57/52	D134G/w	Yes (father and paternal aunt)	/	Ropinirole 14	Asymmetric onset, unilateral left arm rest tremor, bradykinesia and rigidity. Reduced arm swing	Good	1	No	NA	No	No
8	USA	F/59/51	A120S/w^a^	Yes (mother)	NA	NA	Asymmetric onset, bilateral bradykinesia and rigidity. No tremor. Mild gait difficulties	Good	2	No	NA	No	NA
9	Portugal	F/73/17	Q110X/w	Yes (sister; father had tremor)	49	l-DOPA 600 Trihexyphenidyl 6	Bilateral rest and postural tremor (L>R), bilateral rigidity and bradykinesia. Some postural instability	Good	3	No	Urinary urgency	Lower limb dystonia at onset	Marked limb and truncal dyskinesias, off phases in the morning
10	Estonia	M/58/45	Q110E/w	No	55	l-DOPA 400 Entacapone 800 Rasagiline 1 Amantadine 300	Bilateral severe bradykinesia and rigidity, postural instability, mild tremor, hypomimia	Good	3	No	Hyposmia, constipation, fatigue, sleep disorder	No	Mild dyskinesias and wearing-off

NA = information not available; M = male; F = female; PD = Parkinson disease, y = years, ICD = Impulse control disorder, DBS = deep brain stimulation, RBD = REM behavioural sleep disorder, H&Y = Hoehn and Yahr; MMSE = Mini-Mental State Examination.

^a^This case also carries in the heterozygous state the *GBA* E326K variant.

The age at onset of *GCH1*-mutated cases was 43.2 ± 13.4 years (range 17–61). Seven had a positive family history of Parkinson’s disease. DNA of other family members was available for only one case and we showed segregation of the same *GCH1* mutation (Q110X) in the affected sister of the index case. All cases exhibited a variable combination of asymmetrical bradykinesia, rigidity, rest and postural tremor, walking difficulties, postural instability and excellent response to dopaminergic treatment, consistent with a clinical diagnosis of Parkinson’s disease.

The two subjects with the youngest age at onset of symptoms (Cases 4 and 9, who developed symptoms at age 32 and 17, respectively) presented with dystonic features in the lower limbs at onset, a well recognized characteristic of young-onset Parkinson’s disease cases (Bozi and Bhatia, 2003). Case 5 developed lower limb dystonia in off periods over the course of the disease. The remainder did not present with any symptoms or signs of dystonia.

Detailed information about treatment was available for eight cases: the two cases (Cases 1 and 7) with the shortest disease duration (≤5 years) were treated only with a dopamine-agonist, whereas the other cases were taking a combination of levodopa and other anti-parkinsonian drugs. Mean disease duration was 17.6 ± 15.4 years (range 4–56). Cases with longer disease duration displayed a more severe clinical picture with some degree of postural instability (Hoehn and Yahr score ≥ 3), indicating disease progression in spite of the dopaminergic treatment.

In those patients taking levodopa and for whom follow-up information was available (*n* = 7), all developed clinically relevant motor complications of chronic levodopa treatment, including wearing off, motor fluctuations and dyskinesias. Dyskinesias in Case 4 were so disabling that he required treatment with deep brain stimulation of the subthalamic nuclei at age 60.

Most cases exhibited some of the typical non-motor features often recognized in Parkinson’s disease (Lees *et al.*, 2009), such as cognitive difficulties (Case 5), hyposmia (Cases 3–6 and 10) constipation (Cases 4 and 10), urinary problems (Cases 5, 6 and 9), fatigue (Cases 2 and 5) and sleep disturbances (Cases 4–6 and 10).

## Discussion

### Family study

We report here four unrelated DOPA-responsive dystonia pedigrees in which loss-of-function *GCH1* mutations (two splice-site mutations and two missense mutations, confirmed to be pathogenic by metabolic or CSF studies) were found in individuals, asymptomatic for DOPA-responsive dystonia during childhood, who developed adult-onset parkinsonism. They all met the UKPDSBB clinical criteria for a definite diagnosis of Parkinson’s disease and had imaging evidence of a Parkinson’s disease-like nigrostriatal dopaminergic denervation.

A parkinsonian syndrome in the absence of dystonia has been reported in adults who are first-degree relatives of children with DOPA-responsive dystonia. In a series of 21 families, Nygaard showed that 7/50 (14%) individuals older than 40 years had parkinsonism (Nygaard, 1993*a*) and Hagenah *et al.* (2005) reported that 8/23 (34.7%) patients of their series had a positive family history for Parkinson’s disease. *GCH1* mutations have also been shown to segregate in pedigrees with multiple individuals affected by isolated parkinsonism (Irie *et al.*, 2011).

Our study provides evidence that in most of the cases the parkinsonian phenotype in adult *GCH1* mutation carriers is likely due to nigrostriatal degeneration, rather than being simply part of the phenotypic spectrum of metabolic GCH1-related striatal dopamine deficiency. This is consistent with other previous isolated reports of adult-onset parkinsonism in *GCH1* mutation carriers with abnormal nigrostriatal imaging (features summarized in Table 1) (Kikuchi *et al.*, 2004; Hjermind *et al.*, 2006; Eggers *et al.*, 2012; Ceravolo *et al.*, 2013).

Our imaging findings are, however, in apparent contrast to a previous report by Nygaard *et al.* (1992). The authors described a large DOPA-responsive dystonia pedigree, in which three subjects had a late-onset benign parkinsonism, two of which had normal nigrostriatal dopaminergic function determined by means of ^18^F-fluorodopa PET.

Compensatory mechanisms at the presynaptic level (e.g. increased dopamine-intake and dopamine-decarboxylation activity) may result in relatively higher striatal ^18^F-fluorodopa uptake in the initial phase of Parkinson’s disease, underestimating the degree of nigral cell decrease (Nandhagopal *et al.*, 2011). DAT values are therefore a more precise indicator of dopaminergic innervation loss (Ito *et al.*, 1999). We speculate that *GCH1*-parkinsonian cases with normal ^18^F-fluorodopa-PET scan could have upregulated compensatory dopaminergic activity at the presynaptic level, possibly masking the presence of striatal denervation.

In agreement with our findings, Gibb and Lees reported in 1991 a case that presented with juvenile-onset parkinsonism and dystonia with good response to levodopa (commenced at the age of 30) and occurrence of disabling dyskinesias after 1 year of treatment. The patient died at 39 years and pathological examination showed a striking combination of low melanin content in nigral neurons and devastating neuronal loss with reactive gliosis. Furthermore, Lewy bodies were found in surviving nigral cells and in the locus coeruleus (Gibb *et al.*, 1991). This case was subsequently demonstrated to be carrier of a heterozygous mutation in *GCH1* (c.276delC) (Segawa *et al.*, 2004).

### Whole-exome sequencing study

We subsequently showed, in a large cohort of patients with Parkinson’s disease without family history of DOPA-responsive dystonia, that rare *GCH1* coding variants are associated with Parkinson’s disease and increase the disease risk by 7-fold on average.

Among the *GCH1* variants identified by exome sequencing, two (Q110X and K224R) have been shown to cause GCH1 deficiency and DOPA-responsive dystonia in dominant pedigrees (Leuzzi *et al.*, 2002; Saunders-Pullman *et al.*, 2004) and two (V204I and M230I) have been reported in heterozygous sporadic or in recessive cases with DOPA-responsive dystonia (Segawa *et al.*, 2004; Trender-Gerhard *et al.*, 2009; Opladen *et al.*, 2011).

It was not possible to functionally investigate (e.g. phenylalanine-loading test or CSF analysis) the other heterozygous variants identified in this study, therefore their effect on GCH1 activity remains undetermined. However, three of the four novel variants (A120S, D134G and G217V) detected in cases with Parkinson’s disease were located at amino acid positions that are fully conserved through species down to invertebrates and were predicted to be pathogenic by all *in silico* prediction tools, whereas this was not the case for any of the novel mutations present in controls.

Nevertheless, the limitations of prediction tools in reliably distinguishing benign from pathogenic missense changes are well known and therefore we did not exclude any variant from the association test based on predictions scores, possibly underestimating the effect size of *GCH1* pathogenic variants.

Previous studies investigating the contribution of rare coding *GCH1* variants in small cohorts of cases with Parkinson’s disease have reported negative results although these were insufficiently powered to draw conclusions (Bandmann *et al.*, 1996*a*; Hertz *et al.*, 2006; Cobb *et al.*, 2009). An as-yet unpublished meta-analysis of existing genome-wide association study data has, however, identified *GCH1* as a common low-risk locus (Singleton, personal communication), consistent with the hypothesis of a causal role for *GCH1* in Parkinson’s disease.

The mechanism whereby *GCH1* mutations could predispose to nigral cell degeneration is uncertain. Biochemical evidence of GCH1 deficiency and reduced dopamine production has been reported in asymptomatic carriers of *GCH1* mutations (Takahashi *et al.*, 1994; Furukawa *et al.*, 2002). We speculate that GCH1 deficiency and the consequent chronic dopamine deficiency could directly predispose to nigral cell death. This would suggest that normal levels of dopamine exert a protective role on the survival of nigral neurons. There is increasing evidence that levodopa is not toxic to nigral neurons as was previously thought (Parkkinen *et al.*, 2011). Furthermore, activation of dopamine receptors may have a strong anti-apoptotic effect and increase survival of dopaminergic neurons (Nair *et al.*, 2003; Vaarmann *et al.*, 2013). In animal models, levodopa has been shown to promote recovery of nigrostriatal denervation (Datla *et al.*, 2001).

Another possibility is that *GCH1* mutation carriers who do not develop symptoms of DOPA-responsive dystonia in childhood may have compensatory mechanisms that allow for normal nigrostriatal dopaminergic transmission. The maintenance of these mechanisms may increase nigral cell vulnerability to ageing or other environmental and genetic factors, favouring degeneration.

It is also possible that the reduced striatal basal dopamine levels found in *GCH1* mutation carriers may simply lower the threshold of nigral cell loss before parkinsonian symptoms are exhibited. Lastly, we cannot exclude that other yet unrecognized cellular pathways, not related to dopamine synthesis, may be disrupted by GCH1 and BH_4_ deficiency. However, the observation that no DOPA-responsive dystonia cases, treated with levodopa since childhood, have been shown to develop nigral cell loss (Snow *et al.*, 1993; Turjanski *et al.*, 1993; Jeon *et al.*, 1998), supports the notion that levodopa may indeed have a role in reducing the risk of degeneration.

### Limitations of the study

First, dopamine transporter imaging was not available for the cases with Parkinson’s disease with *GCH1* variants identified in the exome sequencing study. It remains a possibility therefore that some of these cases (in particular Case 9, who presented at age 17, with lower limb dystonia and parkinsonism) may represent DOPA-responsive dystonia cases with a parkinsonian phenotype, which may have been misdiagnosed as Parkinson’s disease.

However, removal of the aforementioned case from the statistical analysis did not change substantially the significance of the association (*P* = 0.0003). Furthermore, most of the patients for whom clinical follow-up data were available showed a progressive disease course with increasing levodopa requirements, emergence of motor complications due to chronic treatment with levodopa and presence of classic non-motor features of Parkinson’s disease, strongly supporting nigrostriatal cell loss as the underlying pathology.

Although dyskinesias have been rarely described also in DOPA-responsive dystonia cases, these are significantly different from the ones generally observed in Parkinson’s disease. Indeed they tend to appear at the beginning of the treatment and subside after dose reduction without reoccurring with subsequent slow dose increase (Furukawa *et al.*, 2004; Lee *et al.*, 2013). Second, we could not determine at the individual level the effect on pterin and dopamine metabolism of the *GCH1* variants detected in the exome sequencing study. Reduced penetrance of *GCH1* pathogenic variants for the DOPA-responsive dystonia phenotype is a well-established feature. Nevertheless it has been repeatedly reported, through analysis of brain tissue (Furukawa *et al.*, 2002), CSF (Takahashi *et al.*, 1994) and urine (Leuzzi *et al.*, 2013), that even completely asymptomatic carriers of *GCH1* mutations have abnormal metabolism of biopterins and dopamine, although to a lesser extent than DOPA-responsive dystonia cases. This indicates the existence of a metabolic endophenotype, which we speculate could be the pathogenic mechanism underlying the increased risk for Parkinson’s disease.

Third, we evaluated a cohort enriched with early-onset and familial Parkinson’s disease cases. Thus the frequency of detected *GCH1* variants may not reflect the frequency in late-onset sporadic cases. Finally, we did not assess our samples for the presence of *GCH1* copy number variants, possibly underestimating the frequency of *GCH1* mutations.

## Conclusion

We provide evidence that rare *GCH1* coding variants should be considered as a risk factor for Parkinson’s disease. This is derived both from imaging evidence of striatal dopaminergic denervation in *GCH1* pathogenic variant carriers with a clinical diagnosis of definite Parkinson’s disease (in DOPA-responsive dystonia pedigrees) and from exome sequencing data that show a significant association between *GCH1* coding variants and an increased risk for the disease.

These findings expand the clinical and biological relevance of GCH1 deficiency, suggesting a role not only in biochemical dopamine depletion and DOPA-responsive dystonia, but also in nigrostriatal degeneration. The question as to how the same variants known to cause a Mendelian disease may also exist as risk alleles in Parkinson’s disease may be explained by the well-known reduced penetrance of *GCH1* pathogenic variants. Whether additional genetic or epigenetic factors play a role in determining the clinical phenotype of *GCH1* variant carriers should be addressed by future studies. A better understanding of the relationship between GCH1 deficiency and Parkinson’s disease will shed light on the role of dopamine metabolism on nigral neuron survival, with potential therapeutic implications for patients.

## Supplementary Material

Supplementary Data

## Abbreviations

BH_4_: tetrahydrobiopterin
DAT: dopamine-transporter
^123^I-FP-CIT: [^123^I]N-ω-fluoropropyl-2β-carbomethoxy-3β-(4-iodophenyl) tropane
SPECT: single photon computed tomography
