# Supplementary material for: Parkinson’s disease in GTP cyclohydrolase 1 mutation carriers
Source: Brain. 2014 Jul 2;137(9):2480–92. doi: 10.1093/brain/awu179 (PMC4132650; doi:10.1093/brain/awu179)
Supplement: Supplementary Data [file supp_137_9_2480__index.html]

Parkinson’s disease in GTP cyclohydrolase 1 mutation carriers — Supplementary Data 

# Parkinson’s disease in GTP cyclohydrolase 1 mutation carriers

## Supplementary Data

files

**Files in this Data Supplement:**

- Supplementary Data - doc file
