# Supplementary material for: Parkinson’s disease in GTP cyclohydrolase 1 mutation carriers
Source: Brain. 2014 Jul 2;137(9):2480–92. doi: 10.1093/brain/awu179 (PMC4132650; doi:10.1093/brain/awu179)
Supplement: Supplementary Data [file supp_awu179_brain-2013-02159-File004.doc]

**Supplementary materials**

**RNA analysis performed in family-A**

To test the effect of the splice-site variant c.343+5G>C identified in family-A, messenger RNA (mRNA) was extracted from peripheral blood of individuals II-1 and III-1 using the PAXgene Blood RNA System (Qiagen/PreAnalytix). Following RNA extraction, complementary DNA (cDNA) was synthesized using the Applied Biosystems High-Capacity cDNA reverse transcription kit. The *GCH1* transcript was amplified using exonic primers in exon 1 and exon 3 in both cases and two controls (supplementary Fig. 1A). Following separation by agarose gel electrophoresis, the mutant bands from the patient samples were extracted and sequenced.

Transcript PCR primers

| GCH1_exonic_1F | GGGAGTGTGATCTAAGCAG |
| --- | --- |
| GCH1_exonic_3R | AAGTTTGCTGAGGCCAAGG |

Sequencing of the upper mutant band (352 bp) revealed a mutant transcript whereby exon 2 is spliced to base c.50 of exon 1, splicing-out 293 bp of exon 1 that would be predicted to cause a premature truncation of the protein after 8 out-of-frame amino acids (supplementary Fig. 1A and 1B). Sequencing of the second mutant transcript at ~200 bp failed.

The low intensity of the mutant bands in supplementary Fig. 1A prompted us to consider other possible effects of this mutation. A SYBR Green PCR assay was designed to establish if the c.343+5G>C mutation caused retention of *GCH1* intron 1. Two PCRs were employed, each using the same reverse primer in exon 3 (GCH1_rt_Exon3_R), and a forward primer in either intron 1 (GCH1_rt_Intron1_F) or exon 1 (GCH1_rt_Exon1_F).

rtPCR primers

| GCH1_rt_Intron1_F | catgttactaaagcaagcctctga |
| --- | --- |
| GCH1_rt_Exon1_F | TCTTCACCAAGGGCTACCAG |
| GCH1_rt_Exon3_R | AAGTTTGCTGAGGCCAAGG |

As illustrated in supplementary Fig. 1C, the Intron 1 – Exon 3 PCR captured any transcripts in which at least some splicing had occurred (exon 2 to exon 3), but in which intron 1 had not been spliced-out. This is referred to as 'immature/mutant transcript'. The sequence was confirmed by Sanger sequencing.

The exon 1 – exon 3 PCR captured 'Wild-Type' transcript where exons 1, 2 and 3 were spliced together, also confirmed by sequencing.

PCR reaction mixture: 5 ul 2X SYBR Green Rotor-Gene PCR Master Mix (Qiagen); 10 pmoles each primer; 1 ul cDNA; water to 10 ul. Real-Time PCR was performed on a RotorGene 6000, measuring product accumulation after each amplification cycle: 95°C, 10 minutes; 35 cycles: 95°C, 15 seconds, 60°C,1 minute; Melt curve analysis from 50°C to 95°C. All PCRs for the standard curves and samples were performed in triplicate, the results for sample replicates were averaged. Serial dilutions of control cDNAs were prepared for each PCR over a 27- 490 fold range. Standards and patient samples were amplified on the same experimental run. Rotor-Gene 6000 Series Software 1.7 was used to calculate the cycle threshold (Ct) at which exponential amplification was occurring in the PCRs and a standard curve was plotted for each PCR (log DNA concentration vs. Ct) from the serial dilutions. The relative concentration of each transcript was then calculated for all samples by interpolating from the standard curves. The ratio of immature/mutant transcript : Wild-Type transcript abundance was then derived for each sample.

Supplementary figure 1 RNA analysis performed in family-A


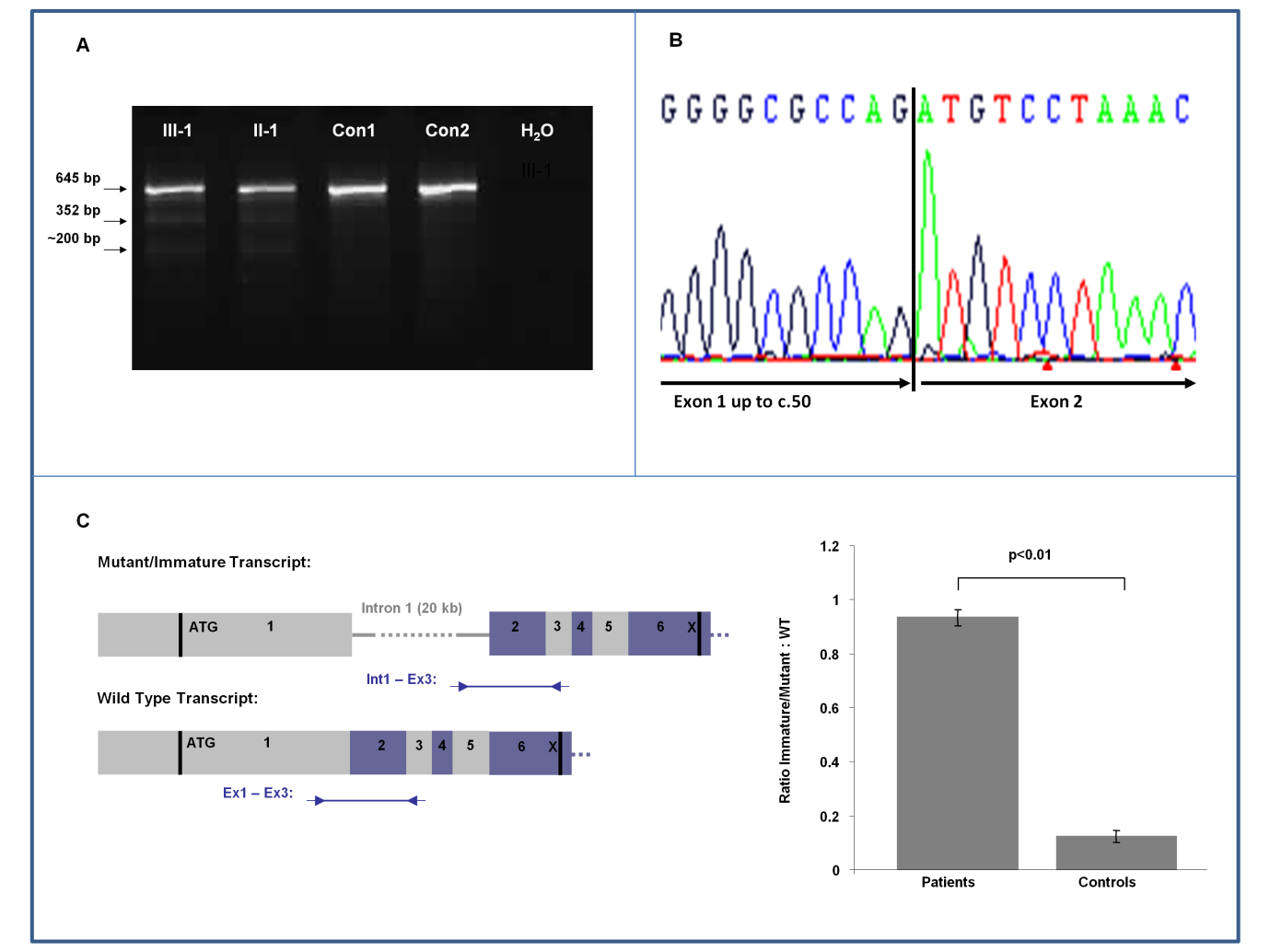


(A) Transcript PCR: PCR from GCH1 exon 1 to exon 3 revealed additional bands in the patients compared to controls (Con1 and Con2) at 352 bp and ~200 bp. Sequencing of the band at ~200 bp failed. (B) Mutant Transcript Sequencing: Sequencing of the 352-bp band revealed splicing of base c.50 to exon 2 (see supplementary data), sequencing of the band at ~200 bp failed. (B) Mutant Transcript Sequencing: Sequencing electropherogram screenshot of the 352 bp band at ~200 bp failed. (B) Mutant Transcript Sequencing: Sequencing electropherogram screenshot showing mutant *GCH1* transcript from patient III-1 whereby exon 2 is aberrantly spliced to c.50 of exon 1. If translated, this would result in 16 in-frame amino acids, followed by 8 out-of-frame amino acids before a premature stop codon. (C) Intron 1 Retention: Two *GCH1* cDNA PCRs each used a common reverse primer in exon 3 and a forward primer either in intron 1 (to detected incompletely-spliced or mutant transcript) or exon 1 (to detect wild-type (WT) transcript). Transcripts are shown with exons 1 to 6 alternately shaded and numbered, the translational start (ATG) and stop (X) sites are indicated with vertical lines. Each SYBR Green PCR was performed on cDNA from patients II-1 and III-1 (two independent extractions from each) and 6 controls. The ratio of the abundance of the transcripts was derived and indicates that the mutant/immature transcript is significantly more abundant relative to WT transcript in the patients than controls (Mean +/- SE; Student's t-test).

**Supplementary figure 2: Phenylalanine-loading test performed in family B**

Abnormally elevated ratios of phenylalanine (Phe) and tyrosine (Tyr) plasma levels at 1, 2, 4, and 6 hours after oral loading with phenylalanine (100mg/kg) in individual I-1 (c.312C>A;p.F104L), II-1 (c.68C>T;p.P23L and c.312C>A;p.F104L) of family B compared to healthy subjects (HS).

**Supplementary table 1: DAT binding values of *GCH1* mutation carriers evaluated in this study.**

| **Subject** | **Phenotype** | **L-putamen** | **R-putamen** | **L-caudatus** | **R-caudatus** |
| --- | --- | --- | --- | --- | --- |
| Family A/ I-1 | PD | 2.08 ^b^ | 2.74 ^a^ | 3.51 | 4.28 |
| Family B/ II-1 | PD | 2.63 ^b^ | 3.73 ^a^ | 4.72 | 4.94 |
| Family B/ I-1 | DRD | 2.85 ^a^ | 2.96 ^a^ | 3.95 | 3.95 |
| Family C/ I-1 | PD | 1.86 ^b^ | 1.64 ^b^ | 2.52 ^b^ | 2.19 ^b^ |
| Family D/ II-1 | PD | 1.53 ^b^ | 2.63 ^b^ | 3.29 ^a^ | 4.5 |
| Family D/ II-2 | DRD | 5.38 | 5.27 | 5.71 | 5.82 |

Values are expressed as the ratio of [^123^I]FP-CIT specific uptake in the region of interest/background. [^123^I]FP-CIT binding values for the caudate nucleus and putamen were calculated by means of the Basal Ganglia Matching Tool.

^a^ Value below 97% inferior confidence limit of healthy controls

^b^ Value below 90% inferior confidence limit of healthy control

**Supplementary table 2: Average exome sequencing coverage of the 6 *GCH1* exons.**

| **Exon** | **Genomic coordinates** | **IPDGC**  **(n=1318)** | **UCL-ex**  **(n=1645)** | **EVS**  **(n=4300)** |
| --- | --- | --- | --- | --- |
| 1 | chr14:55369016-55369405 | 12 (65.4%) | 18 (61.8%) | 6 (59.1%) |
| 2 | chr14:55332045-55332154 | 89 (99.6%) | 48 (87.4%) | 98 (99.7%) |
| 3 | chr14:55326326-55326528 | 58 (99.6%) | 45 (97.1%) | 85 (86.3%) |
| 4 | chr14:55313798-55313934 | 14 (81.8%) | 35 (77.3%) | 20 (83.3%) |
| 5 | chr14:55312427-55312630 | 94 (100%) | 114 (98.9%) | 93 (95%) |
| 6 | chr14:55310683-55310913 | 53 (99.2%) | 22 (80%) | 133 (99.5%) |

IPDGC=International Parkinson Disease Genetic Consortium; UCL-ex= University College of London exomes consortium; EVS= NHLBI Exome Variant Server; chr=chromosome

Data are expressed as the average read depth per region of interest. In brackets is the percentage of samples that had a read depth ≥8 reads.

Coverage of exon 1 was sub-optimal, being the average read depth only 12 in the IPDGC data, 17.9 in the UCL-ex data and 6 in the EVS data. Visual inspections of aligned reads of IPDGC and UCL-ex data (BAM files opened with Genome Browse software, Golden Helix) revealed that the first ~150 coding nucleotides of exon 1 were barely covered (average depth 1-2 reads) in the majority of samples. Similar coverage data were evident for the EVS data (see supplementary figure 3). The coverage of the rest of the exon was good (read depth ≥ 8).

Supplementary figure 3: NHLBI exome variant server sample average coverage depth

**
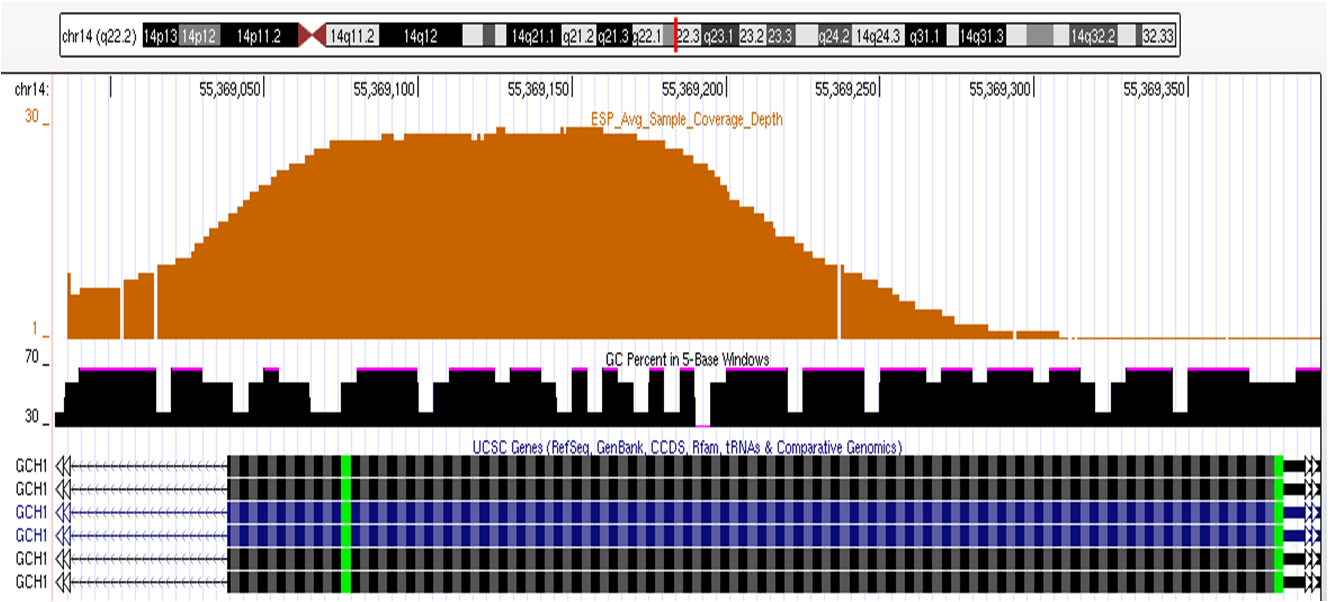
**

Visual representation of coverage depth for GCH1 exon 1 from EVS database. Significantly reduced coverage is evident at the 5’ end of the exon.

**Interspecies alignment of *GCH1* amino acid sequence**

Human ---MEKGPVRAPAEKPRGAR-CSNGF**P**ERDPPRPGPSRPAEKPPRPEAKSAQPADGWKGE 56

Mutant* **L**

Dog ---MEKGPVRAPA-KPRGAR-CSNGF**P**EGEPPRPGPSGPADKPPRPEAKSAQPADGWKGE 55

Mouse ---MEK---------PRGVR-CTNGF**S**ERELPRPGASPPAEKSRPPEAKGAQPADAWKAG 47

Xenopus ---MDPAKSR--PLLEKKAL-NCNGF**L**REDKKEPAAAGSRGDVGR-AGMSTPPVDTWREE 53

Zebrafish ---MERSKQKPVNQSEKETDGAINGH**F**DGRVKMPGWKAGSASGDPGSVPTSSVMESWREE 57

Fruitfly MKPQTSEQNGSGQNGEGAADAVAVAT**I**PTGEASAASATSGTDLTVSKNSQQLKLEMLNLE 60

C.Elegans ---MSRIENESG-------------F**L**SSDAASVGSEDD-------------KVEMK--- 28

Human RPRSEE------------------DNELNL**P**NLAAAYSSILSSLGENPQRQGLLKTPWRA 98

Mutant* **L**

Dog RPRSEE------------------DNELNL**P**NLAAAYSSILRSLGEDPQRQGLLKTPWRA 97

Mouse RHRSEE------------------ENQVNL**P**KLAAAYSSILLSLGEDPQRQGLLKTPWRA 89

Xenopus RARSEE------------------DNELNL**P**SLATAYGTILRALGEDPGRQGLLKTPWRA 95

Zebrafish RTRSLE------------------DNEMSL**P**SIAAAYTTILRGLGEDPQRQGLLKTPWRA 99

Fruitfly LASNGSGHEKCTFHHDLELDHKPPTREALL**P**DMARSYRLLLGGLGENPDRQGLIKTPERA120

C.Elegans -KRNGT-----------------IPKEDHL**K**SMCNAYQSIIQHVGEDINRQGLLKTPERA 70

Human ASAMQ**F**FTKGY**Q**E**T**ISDVLND**A**IFDEDHDEMVIVK**D**IDMFSMCEHHLVPFVGKVH**I**GYLP 158

Mutant* **L** **E A S G** **V**

Dog ATAMQ**F**FTKGY**Q**E**T**ISDVLND**A**IFDEDHDEMVIVK**D**IDMFSMCEHHLVPFVGKVH**I**GYLP 157

Mouse ATAMQ**Y**FTKGY**Q**E**T**ISDVLND**A**IFDEDHDEMVIVK**D**IDMFSMCEHHLVPFVGRVH**I**GYLP 149

Xenopus ATAMQ**Y**FTKGY**Q**E**T**ISDVLND**A**IFDEDHDEMVIVK**D**IDMFSMCEHHLVPFIGKVH**I**GYLP 155

Zebrafish ATAMQ**F**FTKGY**Q**E**K**IIDVLND**A**IFDEDHDEMVIVK**D**IDMFSMCEHHLVPIFGRVH**I**GYLP 159

Fruitfly AKAML**Y**FTKGY**D**Q**S**LEDVLNG**A**VFDEDHDEMVVVK**D**IEMFSMCEHHLVPFYGKVS**I**GYLP 180

C.Elegans AKAMM**A**FTKGY**D**D**Q**LDELLNE**A**VFDEDHDEMVIVK**D**IEMFSLCEHHLVPFMGKVH**I**GYIP 130

Human NKQVLGLSKLARIVEIYSRRLQVQERLTKQIAVAITEAL**R**PAGVG**V**VVEATHMCMVMR**G**V 218

Mutant* **Q I V**

Dog NKQVLGLSKLARIVEIYSRRLQVQERLTKQIAVAITEAL**R**PAGVG**V**VVEATHMCMVMR**G**V 217

Mouse NKQVLGLSKLARIVEIYSRRLQVQERLTKQIAVAITEAL**Q**PAGVG**V**VIEATHMCMVMR**G**V 209

Xenopus NKQVLGLSKLARIVEIYSRRLQVQERLTKQIAIAITEAL**H**PSGVG**V**VVEATHMCMVMR**G**V 215

Zebrafish NKRVLGLSKLARIVEIYSRRLQVQERLTKQIAVAITEAL**Q**PAGVG**V**VVEATHMCMVMR**G**V 219

Fruitfly CNKILGLSKLARIVEIFSRRLQVQERLTKQIAVAVTQAV**Q**PAGVA**V**VVEGVHMCMVMR**G**V 240

C.Elegans NKKVLGLSKLARIVEMFSRRLQVQERLTKQIATAMVQAV**Q**PSGVA**V**VIEASHMCMVMR**G**V 190

Human QKMNS**K**TVTST**M**LGVFREDPKT**R**EEFLTLIRS- 250

Mutant* **R** **I Q**

Dog QKMNS**K**TVTST**M**LGVFREDPKT**R**EEFLTLIKS- 249

Mouse QKMNS**K**TVTST**M**LGVFREDPKT**R**EEFLTLIRS- 241

Xenopus QKMNS**K**TVTST**M**LGVFREDPKT**R**EEFLTLIRS- 247

Zebrafish QKMNS**K**TVTST**M**LGVFREDPKT**R**DEFLTLIRS- 251

Fruitfly QKINS**K**TVTST**M**LGVFRDDPKT**R**EEFLNLVNSK 273

C.Elegans QKINA**S**TTTSC**M**LGVFRDDPKT**R**EEFLNLINKR 223

* Mutant amino acids identified in this study

**IPDGC** **consortium membership**

Mike A Nalls (Laboratory of Neurogenetics, National Institute on Aging, National Institutes of Health, Bethesda, MD, USA), Vincent Plagnol (UCL Genetics Institute, London, UK), Dena G Hernandez (Laboratory of Neurogenetics, National Institute on Aging; and Department of Molecular Neuroscience, UCL Institute of Neurology, London, UK), Manu Sharma (Department for Neurodegenerative Diseases, Hertie Institute for Clinical Brain Research, University of Tübingen, and DZNE, German Center for Neurodegenerative Diseases, Tübingen, Germany), Una-Marie Sheerin (Department of Molecular Neuroscience, UCL Institute of Neurology), Mohamad Saad (INSERM U563, CPTP, Toulouse, France; and Paul Sabatier University, Toulouse, France), Javier Simón-Sánchez (Department of Clinical Genetics, Section of Medical Genomics, VU University Medical Centre, Amsterdam, Netherlands), Claudia Schulte (Department for Neurodegenerative Diseases, Hertie Institute for Clinical Brain Research), Suzanne Lesage (INSERM, UMR_S975 [ formerly UMR_S679], Paris, France; Université Pierre et Marie Curie-Paris, Centre de Recherche de l’Institut du Cerveau et de la Moelle épinière, Paris, France; and CNRS, Paris, France), Sigurlaug Sveinbjörnsdóttir (Department of Neurology, Landspítali University Hospital, Reykjavík, Iceland; Department of Neurology, MEHT Broomfield Hospital, Chelmsford, Essex, UK; and Queen Mary College, University of London, London, UK), Sampath Arepalli (Laboratory of Neurogenetics, National Institute on Aging), Roger Barker (Department of Neurology, Addenbrooke’s Hospital, University of Cambridge, Cambridge, UK), Yoav Ben-Shlomo (School of Social and Community Medicine, University of Bristol), Henk W Berendse (Department of Neurology and Alzheimer Center, VU University Medical Center), Daniela Berg (Department for Neurodegenerative Diseases, Hertie Institute for Clinical Brain Research), Kailash Bhatia (Department of Motor Neuroscience, UCL Institute of Neurology), Rob M A de Bie (Department of Neurology, Academic Medical Center, University of Amsterdam, Amsterdam, Netherlands), Alessandro Biffi (Center for Human Genetic Research and Department of Neurology, Massachusetts General Hospital, Boston, MA, USA; and Program in Medical and Population Genetics, Broad Institute, Cambridge, MA, USA), Bas Bloem (Department of Neurology, Radboud University Nijmegen Medical Centre, Nijmegen, Netherlands), Zoltan Bochdanovits (Department of Clinical Genetics, Section of Medical Genomics, VU University Medical Centre), Michael Bonin (Department of Medical Genetics, Institute of Human Genetics, University of Tübingen, Tübingen, Germany), Jose M Bras (Department of Molecular Neuroscience, UCL Institute of Neurology), Kathrin Brockmann (Department for Neurodegenerative Diseases, Hertie Institute for Clinical Brain Research), Janet Brooks (Laboratory of Neurogenetics, National Institute on Aging), David J Burn (Newcastle University Clinical Ageing Research Unit, Campus for Ageing and Vitality, Newcastle upon Tyne, UK), Gavin Charlesworth (Department of Molecular Neuroscience, UCL Institute of Neurology), Honglei Chen (Epidemiology Branch, National Institute of Environmental Health Sciences, National Institutes of Health, NC, USA), Patrick F Chinnery (Neurology M4104, The Medical School, Framlington Place, Newcastle upon Tyne, UK), Sean Chong (Laboratory of Neurogenetics, National Institute on Aging), Carl E Clarke (School of Clinical and Experimental Medicine, University of Birmingham, Birmingham, UK; and Department of Neurology, City Hospital, Sandwell and West Birmingham Hospitals NHS Trust, Birmingham, UK), Mark R Cookson (Laboratory of Neurogenetics, National Institute on Aging), J Mark Cooper (Department of Clinical Neurosciences, UCL Institute of Neurology), Jean Christophe Corvol (INSERM, UMR_S975; Université Pierre et Marie Curie-Paris; CNRS; and INSERM CIC-9503, Hôpital Pitié-Salpêtrière, Paris, France), Carl Counsell (University of Aberdeen, Division of Applied Health Sciences, Population Health Section, Aberdeen, UK), Philippe Damier (CHU Nantes, CIC0004, Service de Neurologie, Nantes, France), Jean-François Dartigues (INSERM U897, Université Victor Segalen, Bordeaux, France), Panos Deloukas (Wellcome Trust Sanger Institute, Wellcome Trust Genome Campus, Cambridge, UK), Günther Deuschl (Klinik für Neurologie, Universitätsklinikum Schleswig-Holstein, Campus Kiel, Christian-Albrechts-Universität Kiel, Kiel, Germany), David T Dexter (Parkinson’s Disease Research Group, Faculty of Medicine, Imperial College London, London, UK), Karin D van Dijk (Department of Neurology and Alzheimer Center, VU University Medical Center), Allissa Dillman (Laboratory of Neurogenetics, National Institute on Aging), Frank Durif (Service de Neurologie, Hôpital Gabriel Montpied, Clermont-Ferrand, France), Alexandra Dürr (INSERM, UMR_S975; Université Pierre et Marie Curie-Paris; CNRS; and AP-HP, Pitié-Salpêtrière Hospital), Sarah Edkins (Wellcome Trust Sanger Institute), Jonathan R Evans (Cambridge Centre for Brain Repair, Cambridge, UK), Thomas Foltynie (UCL Institute of Neurology), Jing Dong (Epidemiology Branch, National Institute of Environmental Health Sciences), Michelle Gardner (Department of Molecular Neuroscience, UCL Institute of Neurology), J Raphael Gibbs (Laboratory of Neurogenetics, National Institute on Aging; and Department of Molecular Neuroscience, UCL Institute of Neurology), Alison Goate (Department of Psychiatry, Department of Neurology, Washington University School of Medicine, MI, USA), Emma Gray (Wellcome Trust Sanger Institute), Rita Guerreiro (Department of Molecular Neuroscience, UCL Institute of Neurology), Ómar Gústafsson (deCODE genetics and Department of Psychiatry, Oslo University Hospital, N-0407 Oslo, Norway), Clare Harris (University of Aberdeen), Jacobus J van Hilten (Department of Neurology, Leiden University Medical Center, Leiden, Netherlands), Albert Hofman (Department of Epidemiology, Erasmus University Medical Center, Rotterdam, Netherlands), Albert Hollenbeck (AARP, Washington DC, USA), Janice Holton (Queen Square Brain Bank for Neurological Disorders, UCL Institute of Neurology), Michele Hu (Department of Clinical Neurology, John Radcliffe Hospital, Oxford, UK), Xuemei Huang (Departments of Neurology, Radiology, Neurosurgery, Pharmacology, Kinesiology, and Bioengineering, Pennsylvania State University– Milton S Hershey Medical Center, Hershey, PA, USA), Heiko Huber (Department for Neurodegenerative Diseases, Hertie Institute for Clinical Brain Research), Gavin Hudson (Neurology M4104, The Medical School, Newcastle upon Tyne, UK), Sarah E Hunt (Wellcome Trust Sanger Institute), Johanna Huttenlocher (deCODE genetics), Thomas Illig (Institute of Epidemiology, Helmholtz Zentrum München, German Research Centre for Environmental Health, Neuherberg, Germany), Pálmi V Jónsson (Department of Geriatrics, Landspítali University Hospital, Reykjavík, Iceland), Jean-Charles Lambert (INSERM U744, Lille, France; and Institut Pasteur de Lille, Université de Lille Nord, Lille, France), Cordelia Langford (Cambridge Centre for Brain Repair), Andrew Lees (Queen Square Brain Bank for Neurological Disorders), Peter Lichtner (Institute of Human Genetics, Helmholtz Zentrum München, German Research Centre for Environmental Health, Neuherberg, Germany), Patricia Limousin (Institute of Neurology, Sobell Department, Unit of Functional Neurosurgery, London, UK), Grisel Lopez (Section on Molecular Neurogenetics, Medical Genetics Branch, NHGRI, National Institutes of Health), Delia Lorenz (Klinik für Neurologie, Universitätsklinikum Schleswig-Holstein), Alisdair McNeill (Department of Clinical Neurosciences, UCL Institute of Neurology), Catriona Moorby (School of Clinical and Experimental Medicine, University of Birmingham), Matthew Moore (Laboratory of Neurogenetics, National Institute on Aging), Huw R Morris (Department of Clinical Neuroscience, UCL Institute of Neurology, London, United Kingdom), Karen E Morrison (School of Clinical and Experimental Medicine, University of Birmingham; and Neurosciences Department, Queen Elizabeth Hospital, University Hospitals Birmingham NHS Foundation Trust, Birmingham, UK), Ese Mudanohwo (Neurogenetics Unit, UCL Institute of Neurology and National Hospital for Neurology and Neurosurgery), Sean S O’Sullivan (Queen Square Brain Bank for Neurological Disorders), Justin Pearson (MRC Centre for Neuropsychiatric Genetics and Genomics), Joel S Perlmutter (Department of Neurology, Radiology, and Neurobiology at Washington University, St Louis), Hjörvar Pétursson (deCODE genetics; and Department of Medical Genetics, Institute of Human Genetics, University of Tübingen), Pierre Pollak (Service de Neurologie, CHU de Grenoble, Grenoble, France), Bart Post (Department of Neurology, Radboud University Nijmegen Medical Centre), Simon Potter (Wellcome Trust Sanger Institute), Bernard Ravina (Translational Neurology, Biogen Idec, MA, USA), Tamas Revesz (Queen Square Brain Bank for Neurological Disorders), Olaf Riess (Department of Medical Genetics, Institute of Human Genetics, University of Tübingen), Fernando Rivadeneira (Departments of Epidemiology and Internal Medicine, Erasmus University Medical Center), Patrizia Rizzu (Department of Clinical Genetics, Section of Medical Genomics, VU University Medical Centre), Mina Ryten (Department of Molecular Neuroscience, UCL Institute of Neurology), Stephen Sawcer (University of Cambridge, Department of Clinical Neurosciences, Addenbrooke’s hospital, Cambridge, UK), Anthony Schapira (Department of Clinical Neurosciences, UCL Institute of Neurology), Hans Scheffer (Department of Human Genetics, Radboud University Nijmegen Medical Centre, Nijmegen, Netherlands), Karen Shaw (Queen Square Brain Bank for Neurological Disorders), Ira Shoulson (Department of Neurology, University of Rochester, Rochester, NY, USA), Ellen Sidransky (Section on Molecular Neurogenetics, Medical Genetics Branch, NHGRI), Colin Smith (Department of Pathology, University of Edinburgh, Edinburgh, UK), Chris C A Spencer (Wellcome Trust Centre for Human Genetics, Oxford, UK), Hreinn Stefánsson (deCODE genetics), Stacy Steinberg (deCODE genetics), Joanna D Stockton (School of Clinical and Experimental Medicine), Amy Strange (Wellcome Trust Centre for Human Genetics), Kevin Talbot (University of Oxford, Department of Clinical Neurology, John Radcliffe Hospital, Oxford, UK), Carlie M Tanner (Clinical Research Department, The Parkinson’s Institute and Clinical Center, Sunnyvale, CA, USA), Avazeh Tashakkori-Ghanbaria (Wellcome Trust Sanger Institute), François Tison (Service de Neurologie, Hôpital Haut-Lévêque, Pessac, France), Daniah Trabzuni (Department of Molecular Neuroscience, UCL Institute of Neurology), Bryan J Traynor (Laboratory of Neurogenetics, National Institute on Aging), André G Uitterlinden (Departments of Epidemiology and Internal Medicine, Erasmus University Medical Center), Daan Velseboer (Department of Neurology, Academic Medical Center), Marie Vidailhet (INSERM, UMR_S975, Université Pierre et Marie Curie-Paris, CNRS, UMR 7225), Robert Walker (Department of Pathology, University of Edinburgh), Bart van de Warrenburg (Department of Neurology, Radboud University Nijmegen Medical Centre), Mirdhu Wickremaratchi (Department of Neurology, Cardiff University, Cardiff, UK), Nigel Williams (MRC Centre for Neuropsychiatric Genetics and Genomics), Caroline H Williams-Gray (Department of Neurology, Addenbrooke’s Hospital), Sophie Winder-Rhodes (Department of Psychiatry and Medical Research Council and Wellcome Trust Behavioural and Clinical Neurosciences Institute, University of Cambridge), Kári Stefánsson (deCODE genetics), Maria Martinez (INSERM U563; and Paul Sabatier University), Nicholas W Wood (UCL Genetics Institute; and Department of Molecular Neuroscience, UCL Institute of Neurology), John Hardy (Department of Molecular Neuroscience, UCL Institute of Neurology), Peter Heutink (Department of Clinical Genetics, Section of Medical Genomics, VU University Medical Centre), Alexis Brice (INSERM, UMR_S975, Université Pierre et Marie Curie-Paris, CNRS, UMR 7225, AP-HP, Pitié-Salpêtrière Hospital), Thomas Gasser (Department for Neurodegenerative Diseases, Hertie Institute for Clinical Brain Research, and DZNE, German Center for Neurodegenerative Diseases), Andrew B Singleton (Laboratory of Neurogenetics, National Institute on Aging).

**Conflict of interest**

All authors declare no conflict of interest concerning this research.

NEM is funded by a MRC-Wellcome Trust grant. IUI is funded by the Interdisziplinäres Zentrum für Klinische Forschung (IZKF) of the University Hospital Würzburg and the Grigioni Foundation for Parkinson Disease and has received research support from the Grigioni Foundation for Parkinson Disease, the Mariani Foundation for Child Neurology, the Associazione Italiana Rett and the Michael J. Fox Foundation for Parkinson Disease. CG receives grants funded by Actelion, Ipsen, Pharm Allergan and Merz Pharmaceuticals and Academic research support from Deutsche Forschungsgemeinschaft (MU1692/2-1; GA 2031/1-1) and European Science Foundation. AN has received grants from Parkinson UK, Elan/Prothena Pharmaceuticals, GE Healthcare and receives royalties from Radcliffe Publishers. MS has received speaker honoraria from Actelion Pharmaceutical. JHe is funded by a MRC grant. AM receives grants funded by Pharm Allergan, Ipsen, Merz Pharmaceuticals and honoraria for lectures from Pharm Allergan, Ipsen, Merz Pharmaceuticals, Actelion, GlaxoSmithKline and Desitin. He also receives support from non-profit foundations or societies, Possehl-Stiftung, Lübeck, Dystonia Coalition (USA), Tourette Syndrome Association (Germany), European Huntington Disease Network, N.E.MO. (Charity supporting the research of paediatric movement disorders). He receives academic research support from Deutsche Forschungsgemeinschaft (MU 1692/3-1; SFB 936) and Else Kröner-Fresenius-Stiftung. JV is on the Advisory Boards of Medtronic, Boston Scientific, Novartis, has received honoraria from Medtronic, Boston Scientific, Novartis, UCB, TEVA, St. Jude, Abbott and grants from Medtronic, Boston Scientific, Abbott. PK is on the advisory board of Novartis, acts as a consultant for Boston Scientific, received research funding from Novartis, UCB, Orkyn, Aguettant, LVL medical, Teva, Lundbeck, Medtronic, St Jude, and travel cost reimbursement from Abbott, Teva, Novartis, UCB, and Lundbeck. SJL is funded by a MRC grant. HRM is funded by HEFCE, is on the advisory boards of Abbvie, Teva, Boerhinger-Ingelheim, received honoraria for lectures from Teva, UCB, GSK and fundings fro travel from Teva. He received grants funded by Medical Research Council (UK), Wellcome Trust, Parkinson's Uk, Ipsen Fund, Motor Neuron Disease Association, Welsh Assembly Governemnt. He is a co-applicant on a patent based on the C9orf72 mutation (Method for diagnosing a neurodegenerative disease Publication date 2013/3/8 Patent number2013030588). SKo was supported by institutional research funding IUT (IUT20-46) of the Estonian Ministry of Education and Research, by the Centre of Translational Genomics of University of Tartu (SP1GVARENG) and by the European Regional Development Fund (Centre of Translational Medicine, University of Tartu). JHa has received research support from the UK Medical Research Council, the Wellcome Trust, Parkinson's disease UK, and the Brain Research Trust and has consultancies with Eli Lilly, Johnson & Johnson, Eisai, and the UCL Institute of Neurology. SKl is on the advisory board of UCB and receives honoraria from UCB and royalties from Thieme edition. KPB received funding for travel from GlaxoSmithKline (GSK), Orion Corporation, Ipsen, and Merz Pharmaceuticals, LLC; serves on the editorial boards of Movement Disorders and Therapeutic Advances in Neurological Disorders; receives royalties from Oxford University Press); received speaker honoraria from GSK, Ipsen, Merz Pharmaceuticals, LLC, and Sun Pharmaceutical Industries Ltd.; received personal compensation for serving on the scientific advisory boards for GSK and Boehringer Ingelheim; received research support from Ipsen and from the Halley Stewart Trust through Dystonia Society UK, and the Wellcome Trust MRC strategic neurodegenerative disease initiative award (Ref. no,: WT089698), a grant from Parkinson's UK (Ref. no.: G-1009), and a grant from the Dystonia Coalition. NWW has received research support from the UK Medical Research Council, the Wellcome Trust, Parkinson's Disease UK, and the Brain Research Trust. All other authors report no financial disclosures.
